# The effects of land cover change on carbon stock dynamics in a dry Afromontane forest in northern Ethiopia

**DOI:** 10.1186/s13021-018-0103-7

**Published:** 2018-09-06

**Authors:** Negasi Solomon, Opoku Pabi, Ted Annang, Isaac K. Asante, Emiru Birhane

**Affiliations:** 1 Department of Land Resources Management and Environmental Protection, Mekelle University, P.O. Box 231, 7000 Mekelle, Ethiopia; 2 Institute for Environment and Sanitation Studies, University of Ghana, P.O. Box LG 571, 00233 Accra, Ghana; 3 Department of Botany, University of Ghana, P.O. Box LG 571, 00233 Accra, Ghana; 4 Faculty of Environmental Sciences and Natural Resource Management, Norwegian University of Life Sciences, P.O. Box 5003, 1432 Ås, Norway

**Keywords:** Carbon, Carbon dynamics, GIS, Afromontane forest, Land cover, Ethiopia

## Abstract

**Background:**

Forests play an important role in mitigating global climate change by capturing and sequestering atmospheric carbon. Quantitative estimation of the temporal and spatial pattern of carbon storage in forest ecosystems is critical for formulating forest management policies to combat climate change. This study explored the effects of land cover change on carbon stock dynamics in the Wujig Mahgo Waren forest, a dry Afromontane forest that covers an area of 17,000 ha in northern Ethiopia.

**Results:**

The total carbon stocks of the Wujig Mahgo Waren forest ecosystems estimated using a multi-disciplinary approach that combined remote sensing with a ground survey were 1951, 1999, and 1955 GgC in 1985, 2000 and 2016 years respectively. The mean carbon stocks in the dense forests, open forests, grasslands, cultivated lands and bare lands were estimated at 181.78 ± 27.06, 104.83 ± 12.35, 108.77 ± 6.77, 76.54 ± 7.84 and 83.11 ± 8.53 MgC ha^−1^ respectively. The aboveground vegetation parameters (tree density, DBH and height) explain 59% of the variance in soil organic carbon.

**Conclusions:**

The obtained estimates of mean carbon stocks in ecosystems representing the major land cover types are of importance in the development of forest management plan aimed at enhancing mitigation potential of dry Afromontane forests in northern Ethiopia.

**Electronic supplementary material:**

The online version of this article (10.1186/s13021-018-0103-7) contains supplementary material, which is available to authorized users.

## Background

Forest ecosystems are main sources of livelihood for many people and play a crucial role in the economic development of many countries [1, 2]. They are essential natural resources that furnish a wide-range of ecosystem services such as moderating atmospheric carbon balance and thus, climate change [3]. Ecosystem services are the benefits that people get from ecosystem processes which are key to their survival and quality life. Some of these ecosystem services are food, carbon sequestration, nutrient cycling, air and water filtration, and flood amelioration [4]. Carbon sequestration is the capture and storage of carbon that would somehow be produced and kept in the atmosphere or terrestrial systems [5]. Terrestrial systems especially plants represent an important carbon store, estimated globally at 638 Gt, of which 44% is present in plant biomass [6]. Carbon stock varies across forest types. While an average of 303 ton carbon ha^−1^ is retained in tropical forests [7], 66 ton carbon ha^−1^ and 44 ton ha^−1^ are retained in temperate and boreal forests respectively [8].

Ecosystem conditions affect carbon sequestration. Changes in land use including forest clearance for agriculture, settlement and industrial expansion have contributed about 136 (± 55) Gt carbon or one-third of total anthropogenic emissions of carbon dioxide (CO_2_) to the atmosphere over the past 150 years [9, 10]. Carbon emissions from deforestation and forest degradation are the second largest source of anthropogenic carbon emissions [11, 12]. Studies indicate that land cover change has significant effects on carbon stock. For instance, land cover change significantly affected carbon stock by impacting the aboveground biomass and soil organic carbon in Malagasy rainforest, Madagascar [13]. On the other hand, changes in land cover from non-forest to forest ecosystems through exclosure, afforestation and reforestation activities are known to increase the carbon sequestration potential of an area. For example, Mekuria et al. [14] found that the introduction of exclosures on degraded free grazing lands increased carbon stocks in the lowlands of Tigray, Ethiopia. Similarly, Cui et al. [15] indicated that total carbon storage of forest ecosystems increased by approximately 29.3%, from 611.72 Tg in 1993 to 790.75 Tg in 2008, as a result of ecological restoration projects in Shaanxi, Northwest China.

Reducing carbon emissions is of great importance in this era of climate change. Various mechanisms have been proposed by the United Nations Framework Convention on Climate Change (UNFCCC). These include cutting down CO_2_ emissions from Annex 1 countries, and reducing emissions from deforestation and degradation by promoting conservation, sustainable management of forests and enhancing forest carbon stocks (REDD+) [16]. The purpose of REDD+ is to create an incentive for developing countries to protect, better manage and wisely use their forest resources, thereby contributing to the global fight against climate change [17]. One critical element for the REDD+ mechanism is the ability to know the carbon storage potential of a forest ecosystem, and the factors likely to affect both the rate of carbon accumulation and the maximum amount of carbon that can be stored.

REDD+ initiatives have focused on tropical moist forests because of their large carbon stocks per unit area [18] and the substantial emissions of greenhouse gases that would result from converting these forests to pastures, croplands, or commercial timber plantations. Little attention has been paid to the potential for carbon storage and reduction of emissions in the dry forests and woodlands [19–21].

Globally, dry forests cover about 42% of all intra-tropical vegetation [22]. Most of the dry forest ecosystems found in Africa and the world’s tropical islands account for 70–80% of forested areas [23]. Afromontane vegetation cover more than 50% of the land area of the highlands in Ethiopia of which the dry Afromontane forests form the largest part [24]. The Wujig Mahgo Waren state forest is one of the dry Afromontane forests in Ethiopia [25]. The dry Afromontane forests are composed of a number of indigenous tree species dominated by an association of Juniperus-Podocarpus or only Podocarpus species. The forests also contain broad-leaved species such as *Dodonaea angustifolia*, *Carissa spinarum* and *Solanum schimperianum* [26, 27].

The dry Afromontane forests provide a range of ecosystem services including provision of diverse habitats for fauna and fodder for livestock, watershed protection including groundwater regulation, flood control, soil erosion prevention and control, non-timber forest products and climate change mitigation [28–30]. The dry Afromontane forests have not been managed sustainably, and have undergone gradual degradation by human activities over a period of time [31]. However, the Wujig Mahgo Waren forest is one of the remnants of the dry Afromontane forests in northern Ethiopia that continues to provide essential services for the livelihood of the people.

Estimation of changes in ecosystem services, especially carbon stock, due to changes in forest cover have not been of research interest despite its global importance in the face of climate change and REDD+ implementation. Hence, the study (i) quantified carbon stock in different land cover types; (ii) compared the contribution of different carbon pools in different land cover types; (iii) estimated the change in carbon stocks due to forest cover change for the last 30 years; and (iv) evaluated the functional relationship between soil organic carbon stock and aboveground vegetation properties.

## Methods

### Study area

**Fig. 1:**
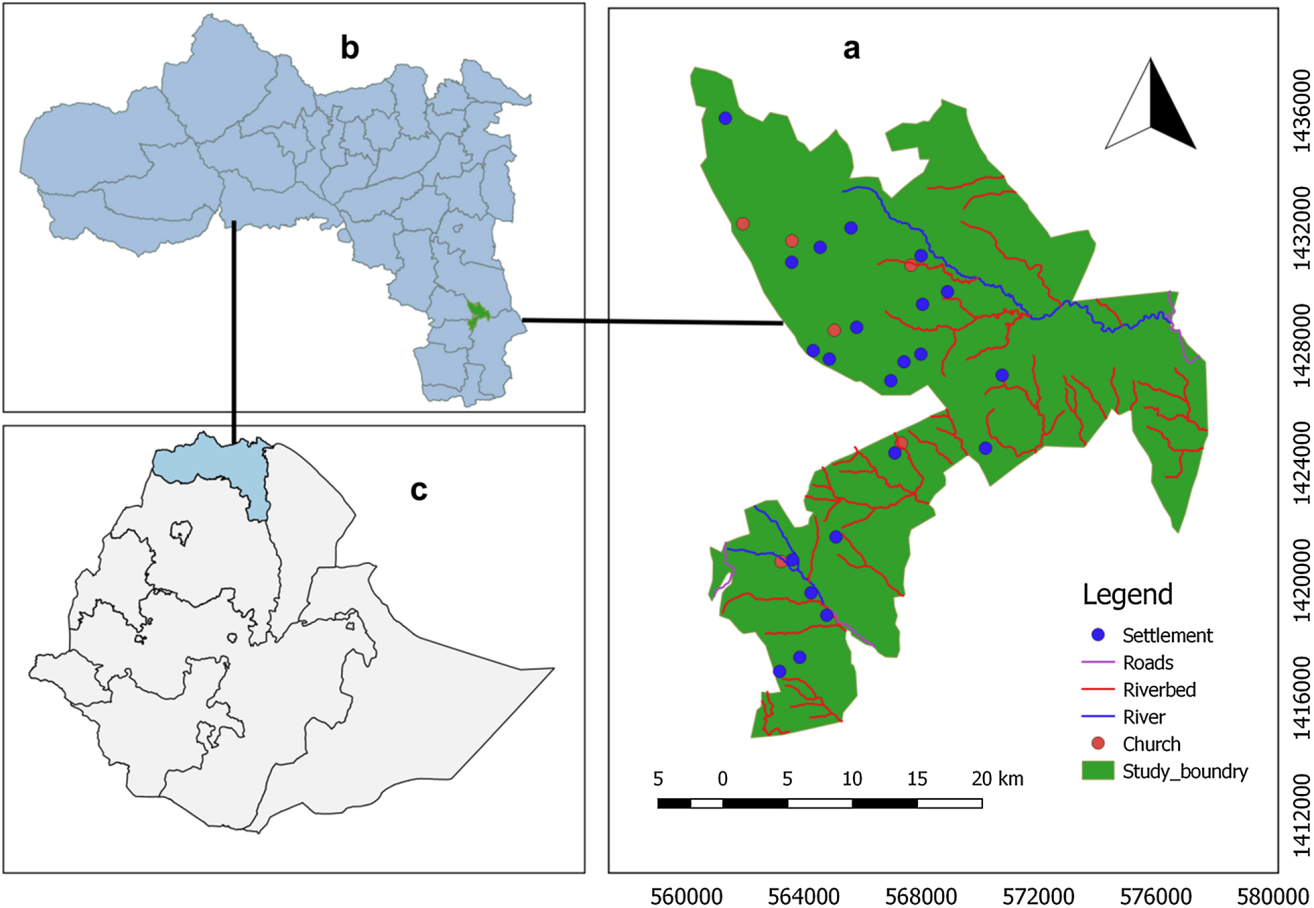
Location map of Wujig Mahgo Waren forest (**a**), Tigray region (**b**) and Ethiopia (**c**)

Wujig Mahgo Waren is located between 12°47′–13°02′ N and 39°26′–39°39′E about 128 km south of Mekelle, the capital city of the Tigray region in northern Ethiopia (Fig. 1). The area has diverse topographic features. A rugged and undulating topography with steep slopes characterizes the landscape. Its elevation ranges from 1404 to 3924 m above sea level. The Wujig Mahgo Waren has a bimodal rainfall distribution pattern. The short and main rainy seasons occur from March to May and July to September, respectively. The area receives an annual rainfall of 833 mm [32]. The mean annual and monthly temperatures range between 8 and 25 °C. The dominant soil types are Vertisols, Cambisols, Fluvisols, Regosols, and Leptosols [32].

The study area covers 17,000 ha. The communities within the study area are engaged in agriculture; the mixed farming system involving crop cultivation and livestock rearing are common. Apart from cultivated lands, the landscape comprises forests, shrub lands, settlements, grassland and bare lands (Table 1). The forest belongs to the tropical dry Afromontane forest type [33]. It is composed of indigenous and exotic species, mainly *Juniperus procera* Hochst. Ex Endl*, Olea europaea* ssp*. africana Mill, Podocarpus falcatus* (Thunb.) Mirb*, Dodonea angustifolia* L.F*., Combretum molle* R.Br. ex G.Don*, Cadia purpurea* (G. Piccioli) Aiton, *Opuntia ficus*-*indica* (L.) Mill, *Acacia abyssinica* [Hochst. ex] Benth, *Eucalyptus globulus* Labill and *Eucalyptus camaldulensis* Dehnh.

**Table 1:** Description of land cover classes used for analysis of change between 1985, 2000 and 2016

Land cover type	Description
Dense forest	All lands with tree cover of canopy density over 40% [34]
Open forest	All lands with tree cover (including mangrove cover) of canopy density between 10 and 40% [34]
Cultivated land	Areas of land prepared for growing agricultural crops. This category includes areas currently under crop and land under preparation
Bare land	Areas with little or no “green” vegetation present due to erosion, overgrazing and crop cultivation
Grassland	Lands covered by herbaceous plants with coverage greater than 5% and land mixed rangeland with the coverage of shrub canopies less than 10% [35]. Among the herbaceous species, *Cynodon dactylon* and *Pennisetum petiolar* had greater frequencies in the study area

### Vegetation, litter and soil sampling

The vegetation, litter and soil samples of the forest were quantified using a systematic sampling design. Ten parallel line transects with 1 km distance were laid throughout the forest. Randomly selected 20 m × 20 m sample plots (main plots) were demarcated for trees and shrub assessment, and five 1 m × 1 m subplots within the main plot designated for litter and soil sampling. There were 88 sample plots set at 400 m intervals along transects.

The number of main plots were determined using Pearson et al. [36] equation;

$${\text{n}} = \frac{{\left( {\mathop \sum \nolimits_{{{\text{i}} = 1}}^{\text{n}} {\text{Ni}}*{\text{Si}}} \right)^{2} }}{{\frac{{{\text{N}}^{2} *{\text{E}}^{2} }}{{{\text{t}}^{2} }} + \left( {\mathop \sum \nolimits_{{{\text{i}} = 1}}^{\text{n}} {\text{Ni}}*{\text{Si}}^{2} } \right)}}$$

where E = allowable error or the desired half-width of the confidence interval. Calculated by multiplying the mean carbon stock by the desired precision (that is, mean carbon stock × 0.1, for 10% precision), t = the sample statistic from the t-distribution for the 95% confidence level; *t* is usually set at 2 as the sample size is unknown at this stage, N_i_ = number of sampling units for land cover type *i* (= area of land cover type in hectares), n = number of sampling units in the population, s_i_ = standard deviation of land cover *i*.

All trees and shrubs were identified in the plots. A botanist supported by the local people was engaged to confirm scientific names and local names of the plant species. Diameter at breast height (DBH) and height (H) of all trees and shrubs with DBH ≥ 2 cm were measured using measuring tape and a 5 m pole graduated with 10 cm markings respectively from each main plot. Trees taller than 5 m were measured using clinometer positioned at 10 m distance from the base of the tree and focused on the highest point of the tree. Litter samples were collected from five 1 m × 1 m subplot within the main plot. A composite sample of 100 g was placed in a plastic bag and taken to the laboratory for litter carbon analysis.

Soil samples were collected from five subplots within main plot at a depth of 30 cm using a core sampler. All samples were placed in paper bags with appropriate labels. A composite sample of 100 g from each plot was submitted to analyze bulk density and soil organic carbon.

### Land cover data

The land cover data were obtained from Solomon et al. [33] (Table 2). The datasets were generated by employing supervised classification of Landsat satellite images. They included area statistics of five different land cover types for the year 1985, 2000 and 2016.

**Table 2:** Area and proportion of land cover (LC) in Wujig Mahgo Waren forest in 1985, 2000, and 2016

LC types	Land cover distribution					
	1985		2000		2016	
	Area (ha)	%	Area (ha)	%	Area (ha)	%
Dense forest	4469	26	4836	28	4335	25
Open forest	3629	21	4802	28	4337	25
Grassland	1713	10	1074	6	2035	12
Cultivated land	3211	19	3035	18	3902	23
Bare land	3999	24	3272	19	2417	14

### Carbon quantification

#### Biomass carbon stock assessment

Aboveground biomass (AGB) was estimated using the equation of Chave et al. [37] provided below.

$${\text{AGB }}({\text{kg}}) = 0.0673*(\uprho{\text{DBH}}^{2} {\text{H}})^{0.976}$$

where, DBH is diameter at breast height, H is total tree height and ρ is wood specific gravity = 0.58 g cm^−3^ the arithmetic mean for tropical Africa. This equation was developed for similar agroecologies that represent the study area. Specific allometric equations for aboveground biomass were used for species that have allometric equation (Table 3).

**Table 3:** Allometric equations used for aboveground biomass calculation

Woody species	Dependent variable	Allometric equation	Unit	*r* ^*2*^	References
*Juniperus procera*	AGB	AGB = 1.12 × DBH^1.54^	kg	0.95	[29]
*Acacia abyssinica*	AGB	AGB = 0.55 × DBH^1.89^	kg	0.97	[29]
*Acacia etbaica*	TDW	Ln totWt = 2.11 + 2.19 × LnDSH	kg	0.96	[39]
*Euclea shimperi*	TDW	Y = 63.07 × DSH^1.78^	g	0.95	[40]
*Otostegia integrifolia*	TDW	Y = 45.80 × DSH^2.26^	g	0.99	[40]
*Other shrub* sps.	TDW	Y = (0.3197 × DSH) + (0.0383 × DSH^2.6^)	kg	0.93	[41]

Belowground biomass (BGB) was estimated using the regression model given by Cairns et al. [38]:

$${\text{BGB }}({\text{kg}}) = \exp ( - 1.0580.8836\ln {\text{AGB}})$$

where, AGB = aboveground biomass density.

The conversion of biomass to carbon stocks was done using Pearson et al. [36]. According to this equation, 50% of the measured biomass is carbon.

$${\text{Carbon }}{\kern 1pt} ({\text{kg}}) = 0.5*{\text{biomass}}$$

#### Litter carbon estimation

To estimate litter carbon 100 g of composite fresh weight of litter was collected from the five-subplot sample and oven dried at 105 °C. Litter biomass was estimated using Pearson et al. [36] equation.

$${\text{Dry mass}} = \left[ {\frac{\text{dry mass of composite sample}}{\text{fresh mass of composite sample}}} \right]*{\text{X}},$$

where X is total fresh mass of whole sample

Litter carbon stock was estimated as:

$${\text{Litter carbon }}\,({\text{Mg ha}}^{ - 1} ) = {\text{dry mass}}*{\text{\% carbon}}$$

Percentage of carbon is the carbon fraction of IPCC with a default value of 0.37.

#### Soil carbon stock assessment

Soil organic carbon (SOC) was calculated using Pearson et al. [36].

$${\text{Soil organic carbon}} = {\text{bulk density}}*{\text{depth}}*{\text{\% carbon}}$$

where,

$${\text{Bulk density }}({\text{g cm}}^{ - 3} ) = \frac{{{\text{oven dry mass }}\left( {\text{g}} \right)}}{{{\text{volume }}\left( {{\text{cm}}^{3} } \right)}}$$

%Carbon = Carbon concentration (%) determined in the laboratory following Walkley and Black [42] method.

#### Total carbon stock

The total carbon stock (C_t_) was the summation of the carbon stock values of the individual carbon pools of the land cover type.

$${\text{C}}_{\text{t}} ({\text{Mg ha}}^{ - 1} ) = {\text{AGC}} + {\text{BGC}} + {\text{LC}} + {\text{SOC}}$$

where, AGC = above ground carbon stock, BGC = belowground carbon stock, LC = litter carbon stock and SOC = soil organic carbon.

The carbon stocks for 1985 and 2000 were obtained by assuming that individual cover class carbon values did not change [43–45].

### Carbon mapping

Mapping of carbon stock by exponential semivariogram model was done to estimate spatial distribution of carbon values [46].

### Soil texture analysis

Soil texture analysis was performed using the hydrometer method.

### Statistical analysis

The SAS 9.0 was used to perform one-way analysis of variance (ANOVA) to test for mean differences of vegetation parameters, carbon stock means across land covers and carbon pools. Tukey HSD test was performed to separate means.

The Minitab computer statistical software was used to perform multiple linear regression analyses on soil organic carbon stock, biomass carbon stock, average tree diameter, and average tree height and tree density. The stepwise multiple regression with backward and forward selection techniques was used to select predictor variables.

## Results

### Vegetation characteristics

The total number of trees identified in the forest were 3290. The trees belong to 29 families. Forty five woody species were recognized in all land cover categories on all plots (Additional file 1). *Cadia purpurea*, *Dodonaea angustifolia*, *Maytenus arbutifolia, Juniperus procera*, *Calpurnia aurea, Carissa spinarum and Acacia abyssinica* were the seven dominant species that contributed 72% of the total species abundance. *Cupressus lusitanica*, *Eucalyptus camaldulensis* and *Eucalyptus globlus* are exotic species, while the remaining species are native. The forest had an overall tree density of 1158 ± 74 stems ha^−1^ (mean ± SE). The stem density of the dense forest (1618.3 ± 93.4 ha^−1^) was significantly higher than the density of the open forest (959.1 ± 64.9 ha^−1^) and grassland (196.9 ± 19.7 ha^−1^). The average stem diameter differed across land cover categories, with 7.21 ± 0.51 cm being for the dense forest, 5.56 ± 0.47 cm for the open forest and 2.96 ± 0.172.8 cm for the grassland (Table 4). Average woody plant height of all species was higher in dense forest (4.3 ± 0.44 m) followed by open forest (3.03 ± 0.21 m) and grassland, 1.89 ± 0.10 m (Table 4).

**Table 4:** Average (± standard error) woody plant dendrometric variables and average number of stems under different land cover types

Land cover type	DBH (cm)	H (m)	# of stems ha^−1^
Dense forest	7.21 ± 0.51^a^	4.3 ± 0.44^a^	1618.3 ± 93.4^a^
Open forest	5.56 ± 0.47^b^	3.03 ± 0.21^b^	959.1 ± 64.9^b^
Grass land	2.96 ± 0.17^c^	1.89 ± 0.10^b^	196.9 ± 19.7^c^
*p* value	0.0003	0.001	< 0.0001

Values within a column with same letters are not significantly different (p > 0.05) according to Tukey’s HSD test

*DBH* diameter at breast height, *H* height

### Soil characteristics

The soil physical properties and soil organic carbon varied with land use type (Table 5). The dense forest recorded higher values for soil organic carbon concentration than the other land-use types. Organic carbon ranged between 2.0 and 3.1%, with the highest occurring in dense forest, and lowest on bare land. In the dense forest, soils were higher in clay content than in the open forest, and the mean value for bulk density of the soil varied from 1.11 to 1.37 g cm^−3^: with the highest content in bare land and lowest in dense forest.

**Table 5:** Average (± standard error) soil properties (0–30 cm) of different land uses in the Wujig Mahgo Waren forest of Ethiopia

Land uses	Particle size distribution			OC (%)	BD (g cm^−3^)
	Sand (%)	Silt (%)	Clay (%)		
Dense forest	33.1 ± 3.3^ab^	34.5 ± 2.9^a^	32.3 ± 2.3^a^	3.1 ± 0.17^a^	1.11 ± 0.05^a^
Open forest	27.2 ± 2.6^ab^	43.8 ± 2.2^a^	28.0 ± 1.6^a^	2.7 ± 0.16^a^	1.17 ± 0.04^a^
Grassland	29.2 ± 8.1^ab^	42.5 ± 7.1^a^	28.3 ± 2.6^a^	2.8 ± 0.27^a^	1.28 ± 0.07^a^
Cultivated land	19.7 ± 3.6^b^	48.4 ± 4.4^a^	31.8 ± 3.2^a^	2.2 ± 0.33^a^	1.31 ± 0.04^a^
Bare land	50.2 ± 11.1^a^	27.8 ± 8.0^a^	22.0 ± 6.0^a^	2.0 ± 0.25^a^	1.37 ± 0.05^a^
*p*-value	0.023	0.02	0.23	0.06	0.035

Values within a column with same letters are not significantly different (p > 0.05) according to Tukey’s HSD test

*OC* soil organic carbon, *BD* bulk density

### Carbon stocks

The mean biomass carbon stock was five times higher in the dense forest compared to the open forest and twenty times higher than that of the grassland (Table 6). The above and below ground carbon stock was not significantly different between the open forest and grassland (Table 6). The carbon concentrations were highly influenced by land use (Table 6).

**Table 6:** Estimated carbon stocks (Mg ha^−1^) across the land cover types

C contents of	Dense forest	Open forest	Grassland	Cultivated land	Bareland	*p*-value
agb	65.81 ± 18.50^a^	12.67 ± 2.22^b^	3.43 ± 0.33^b^	–	–	< 0.001
bgb	11.38 ± 2.61^a^	2.92 ± 0.41^b^	1.02 ± 0.08^b^	–	–	< 0.0002
Lb	2.25 ± 0.27^a^	1.68 ± 0.20^ab^	1.17 ± 0.09^b^	–	–	< 0.0048
SOC	102.33 ± 13.2^a^	87.55 ± 12.73^a^	103.13 ± 6.75^a^	76.54 ± 7.84^a^	83.13 ± 8.53^a^	< 0.271
Total	181.78 ± 27.1^a^	104.83 ± 12.35^b^	108.77 ± 6.77^b^	76.54 ± 7.84^b^	83.11 ± 8.53^b^	< 0.0001

Values within a row with same letters are not significantly different (p > 0.05) according to Tukey’s HSD test

*agb* above ground biomass, *bgb* belowground biomass, *Lb* litter biomass, *SOC* soil organic carbon

The carbon content of litter biomass was significantly higher under dense forest than grassland (Table 6). The mean litter carbon was high in open forest as compared to grassland. Soil organic carbon was higher in grassland and the lowest mean soil organic carbon was recorded in cultivated land (Table 6). The conversion of dense forests to cultivated land resulted in a 25% reduction in soil organic carbon stock.

**Fig. 2:**
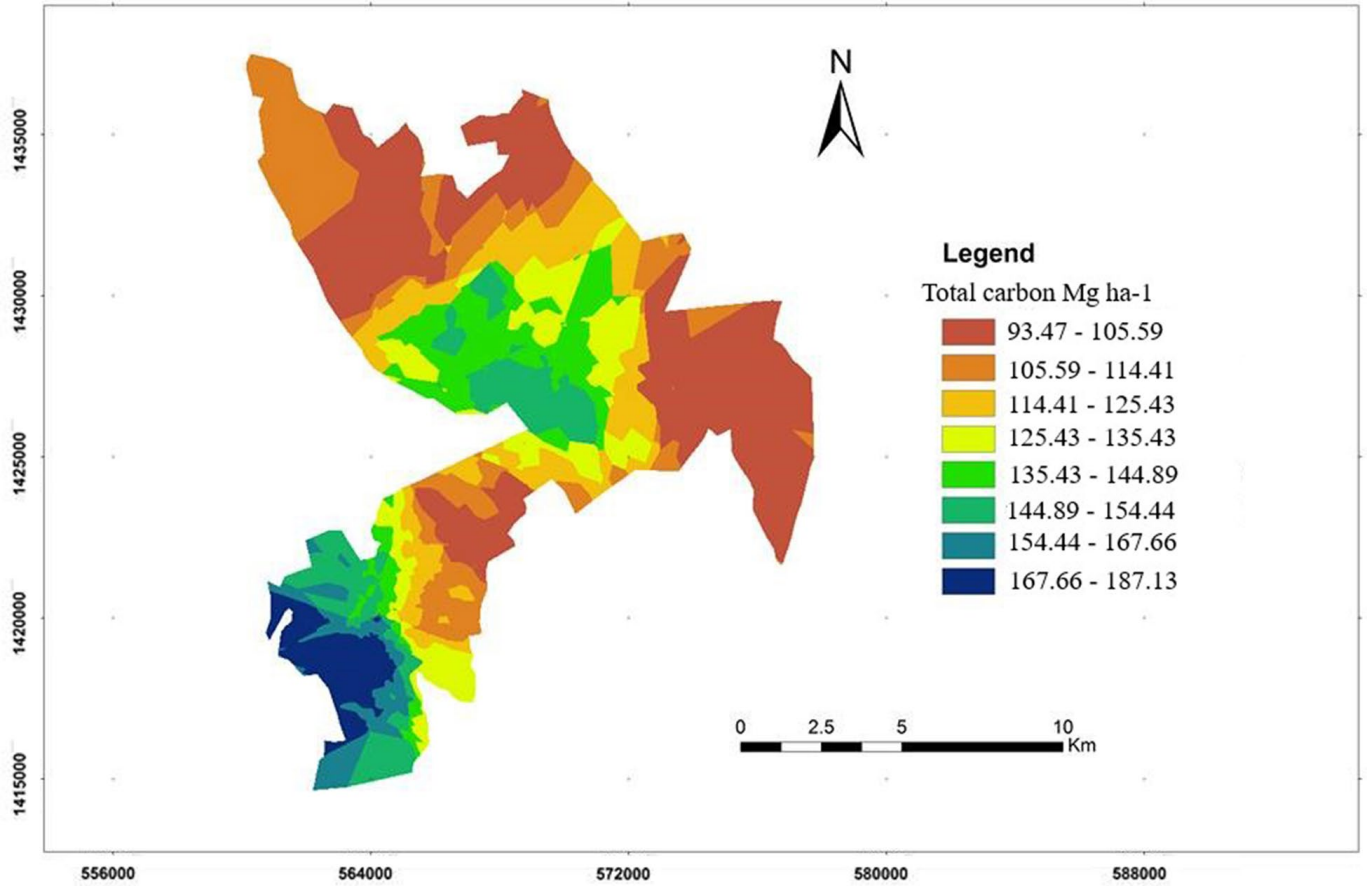
Spatial distribution of total carbon stock (Mg ha^−1^) in the study area in 2016

The estimated total carbon stock density was high in dense forest and low in cultivated land and bare land cover while open forest and grassland sites showed intermediate values. Total ecosystem carbon stock ranged from 76.54 ± 7.84 to 181.78 ± 27.06 Mg ha^−1^ in the following order: dense forest > grassland > open forest > bare land > cultivated land (Table 6 and Fig. 2; Additional file 1). The soil contributed the higher carbon stock to the total carbon stock of grassland, cultivated land and bare land.

### Contribution of carbon pools

The relative importance of the different carbon pools varied with the land cover classes. Soil organic carbon and aboveground carbon stock contributed more to the overall carbon stocks across the land uses (Table 7). For example, soil carbon accounted for 100% of total carbon stocks for agriculture and bare land (Table 7). In this study, all data demonstrate that carbon stored in the soil pool was higher than the carbon stored in the biomass. In the dense forest, 56.3% and 36.2% of the total carbon stock was stored in soil and aboveground biomass, respectively. The remaining carbon was stored in belowground biomass and litter. Similarly, in the open forest, significantly higher carbon stock was stored in soil (83.5%) followed by aboveground carbon (12%), belowground carbon (2.8%) and litter carbon (1.1%). Carbon stocks of litter were not significant compared to those in biomass and soil. In the grassland, a large proportion (> 90%) of total ecosystem carbon was stored in the soil (Table 7).

**Table 7:** Carbon stocks (Mg ha^−1^) in different carbon pools in Wujig Mahgo Waren forest

Land uses	agC	bgC	SOC	Lc	*p*-value
Dense forest	65.81 ± 18.50^a^	11.38 ± 2.61^b^	102.33 ± 13.19^a^	2.25 ± 0.27^b^	< 0.0001
Open forest	12.67 ± 2.22^b^	2.92 ± 0.41^b^	87.55 ± 12.73^a^	1.68 ± 0.20^b^	< 0.0001
Grassland	3.43 ± 0.33^b^	1.02 ± 0.08^b^	103.13 ± 6.75^a^	1.17 ± 0.09^b^	< 0.0001

Values within a row with same letters are not significantly different (p > 0.05) according to Tukey’s HSD test

*agC* above ground carbon, *bgC* belowground carbon, *LC* litter carbon, *SOC* soil organic carbon

### Effect of land cover change on carbon stocks

**Fig. 3:**
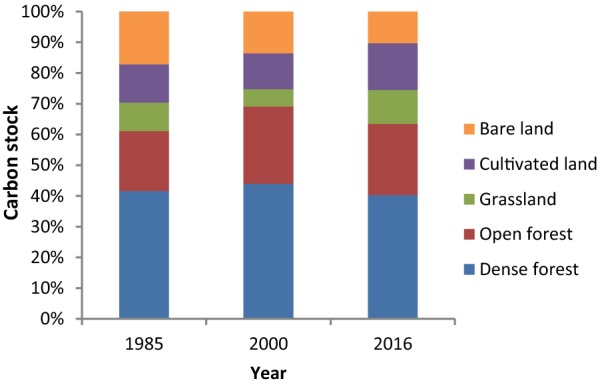
Estimated carbon stock for each land cover type of the different reference years of the study area

In the first study period (1985–2000), carbon stock slightly increased (Fig. 3). However, in the second study period (2000–2016), a decrease in carbon stock was observed. There was a net increase in carbon stock throughout the entire study period studied.

The total carbon stock in 2000 was higher than in 1985 and 2016. In 1985, 2000 and 2016, the carbon stock found in Wujig Mahgo Waren was 1951 Gg carbon, 1999.81 Gg carbon and 1955.63 Gg carbon, respectively (Table 8). In the dense forest, total carbon stock was higher in 2000 followed by 1985 and 2016, respectively. Similarly, in the open forest, the highest carbon stock was recorded in 2000 followed by 2016 and 1985, respectively. However, in the grassland, the highest carbon stock was recorded in 2016. Similarly, in cultivated land, the highest carbon stock was recorded in 2016. In the bare land carbon stock was highest in 1985.

**Table 8:** Total carbon stock (Gg) for Wujig Mahgo Waren forest in the year 1985, 2000 and 2016

Land cover types	Carbon stocks (Gg)			Carbon stock changes (Gg)		
	1985	2000	2016	1985–2000	2000–2016	1985–2016
Dense forest	812.32	879.15	787.95	66.83	− 91.2	− 24.37
Open forest	380.42	503.44	454.09	123.02	− 49.35	73.67
Grassland	180.20	112.97	214.10	− 67.23	101.13	33.9
Cultivated land	245.74	232.29	298.62	− 13.45	66.33	52.88
Bare land	332.32	271.96	200.87	− 60.36	− 71.09	− 131.45
Total	1951.00	1999.81	1955.63	48.81	− 44.18	4.63

### Relationship between soil organic carbon (SOC) stock and aboveground vegetation properties

#### Correlations between SOC stock and vegetation parameters

Table 9 presents the Pearson correlation values between vegetation parameters and soil organic carbon stock. The highest statistically significant correlations were found between DBH and SOC (Pearson correlation 0.63, p < 0.01), followed by H and SOC (Pearson correlation 0.5, p < 0.05). The lowest correlation was found between tree density and DBH (Pearson correlation − 0.042, p > 0.05).

**Table 9:** Pearson correlation coefficient values of soil organic carbon, DBH, height and tree density

	SOC	DBH	H	Tree density
SOC	1.00			
DBH	0.627**	1.00		
H	0.502*	0.107	1.00	
Tree density	0.437	− 0.042	0.457	1.00

*p < 0.05; **p < 0.01

#### Regression models of soil organic carbon stock

Links between soil organic carbon stock and aboveground vegetation properties remained significant (Table 10), indicating that vegetation properties do seem to be general predictors of soil organic carbon stock. The multiple regression analysis indicated that the best-fit model, based on the Akaike Information Criteria (AIC) predicts soil organic carbon as a function of tree density, DBH and height (Table 10). The results of the analysis also indicated that DBH is the most significant predictor of SOC (p = 0.00332).

**Table 10:** Regression model of soil organic carbon stock in Wujig Mahgo Waren forest

Dependent variable	Term	Coefficient	Adj. R^2^	p	AIC
SOC	Intercept	− 11.6	0.59	0.003	104.04
	Tree density	0.01			
	DBH	7.22			
	Height	3.96			

**Fig. 4:**
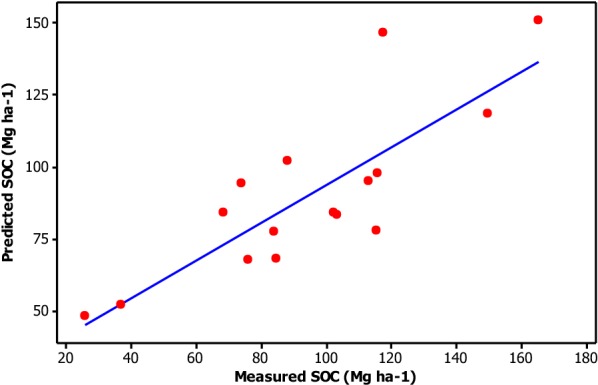
Predicted vs. measured soil organic carbon stock

The observed mean soil organic carbon was 95 ± 9.0 whereas the predicted soil organic carbon was 90.5 ± 7.2. The observed and predicted soil organic carbons were similar (Fig. 4; Additional file 1).

## Discussion

### Effects of land cover change on carbon stock

The study showed how carbon stocks in vegetation, litter and soils were varied across land cover types and different periods. Dense forests had higher biomass carbon stock compared to open forests and that of the grassland. Rajput et al. [47] and Solomon et al. [29] found higher biomass carbon in forest ecosystems as compared to other land cover types in northwestern Himalaya and northern Ethiopia, respectively. The substantial variation in biomass carbon across the land cover types might be due to the variation in the number of stems, density and the size of the trees in each land cover type. This is in line with the result of Solomon et al. [29] which stated that tree density and diameter have an effect on biomass carbon in northern Ethiopia. Moreover, the low biomass carbon recorded in grasslands was caused by overgrazing practices and human intrusion that influenced the recovery and growth of herbaceous plant species and adversely smothers tree and shrub growth [33]. This assertion is supported by the study conducted by Mekuria and Yami [48] who suggested that free grazing affects vegetation composition and growth of herbaceous plant species in the drylands of northern Ethiopia.

The biomass carbon estimates of the dense forests were within the global range, from 20 to 150 Mg ha^−1^ for semiarid tropics as reported by Tiessen et al. [49]. The results were also within the range of tropical dry forests’ carbon stock [50] which was between 50 and 350 t ha^−1^. However, the average biomass carbon stock of Wujig Mahgo Waren forest was lower than the Egdu forest [51] which was 337 t ha^−1^ found in similar agroecology. The biomass carbon stock of the present study was fairly small compared to the biomass carbon stocks in the moist Bale forest in Ethiopia [52]. On the other hand, the biomass carbon stock in the current study was fairly higher compared to Solomon et al. [29] who reported 58.11 Mg ha^−1^ in the managed forest of Tigray, northern Ethiopia. As compared to the present study, Chinasho et al. [53] found lower carbon stock with 45.23 t ha^−1^ in woody plants of Humbo forest, southern Ethiopia. The variability in biophysical characteristics such as climate, soil and vegetation type might contribute to the difference in biomass carbon stock across the different forests.

The carbon content of litter biomass was significantly higher under dense forests than grasslands. The difference in litter carbon among the land cover types might be due to the variations in vegetation cover. This was confirmed by the study of Descheemaeker et al. [54] who stated that litter accumulation rely upon vegetation cover and is affected by soil fertility in exclosures of the Tigray highlands, Ethiopia. The estimated litter carbon of the present study is in accordance with findings reported by Ordóñez et al. [55], who found between 0.6 and 4.1 Mg ha^−1^ of litter carbon in montane forests of central and southern Mexico. However, the estimated value of litter carbon in the present study was higher than that reported by Aman [56] who found 1.38 t ha^−1^ litter carbon in dry evergreen montane forests of the Bale mountain national park, Ethiopia. Conversely, compared to the litter carbon stocks of Chilimo forest (9.36 Mg ha^−1^) per Tesfaye et al.’s [57] observation, the current result was very low.

There was higher soil organic carbon stock in grassland and dense forest as compared to open forest, bare land and cultivated land. The differences recorded in soil organic carbon between land cover types were not significant. In agreement with the present study, Haghdoost et al. [58] showed that no significant difference existed in the average total soil carbon stock among land cover types in Noor county, Iran, though higher soil carbon was found in forests as compared to cultivated lands. Ordóñez et al. [55] also found no significant difference in average total soil carbon in the central highlands of Michoacan, Mexico. The higher mean soil organic carbon stock in grassland compared with the other land uses could be due to higher annual turnover of organic matter from dying grassroots. This notion was supported by the report of Guo and Gifford [59], who stated that grassroots decompose faster than tree roots and hence contribute higher organic matter to soils. The higher soil organic carbon stock recorded in the dense forest was mainly because of the biomass inputs and low rate of litter decay. Tesfaye et al. [57] also found a higher mean carbon stock in natural forest than in all the other land cover categories in Chilimo, a dry Afromontane forest in Ethiopia. The lower soil organic carbon recorded in the cultivated land might be due to the low input of organic matter being returned to the soil and high rates of oxidation of soil organic matter by tillage [60].

The high carbon content of the soils in the different land cover types was consistent with a previous study by Lemenih and Itanna [61] who studied soil carbon stock for the upper 60 cm depth of soil in southern Ethiopia. The result of this study were also within the ranges of values for tropical soils of 86 Mg carbon ha^−1^ [62], 113 Mg carbon ha^−1^ [63] and 72.8–116.4 Mg carbon ha^−1^ of montane forests of Central Highlands of Michoacan, Mexico [55]. Contrary to the results of this current study, Feyissa et al. [51] found higher soil organic carbon in Egdu Forest, Ethiopia. On the other hand, higher soil organic carbon stock was recorded under the present study as compared to the results reported by Girmay and Singh [64] for Maileba and Gum Selassa sites of northern Ethiopia.

Land cover change can change soil carbon stock. The results indicated that alteration of dense forests to cultivated land brought about 25% reductions in soil organic carbon stock. Girmay et al. [65] who reviewed carbon stock in top soils (0–10 cm) of Ethiopia, found that conversion of native forest into croplands and plantations reduced carbon stock by up to 63% and 83%, respectively.

Generally, dense forest had higher total carbon stock followed by open forest, grassland, cultivated land and bare land in this study. The average total carbon stock of the dense forest was 181.8 Mg ha^−1^, which was higher than that reported by Mekuria [66] for exclosures on communal grazing lands in Ethiopia. Similarly, the results were slightly higher than that reported by Andriamananjara et al. [13] for the Malagasy rainforest in eastern Madagascar. The carbon stock in the present study was lower than the carbon stock for Northwestern Himalaya [47], for Egdu forest [51], for montane forests of central and southern Mexico [55] and for low land area of Simien mountains national park [67]. The variations in total carbon stock among the different studies might be due to variation in forest composition, soil and other biophysical factors.

In this study, the four carbon pools contributed differently to the five land cover classes. Higher levels of carbon were stored in the soil pool rather than the vegetation biomass and litter carbon of all land cover types. Most of the carbon stocks in grassland, cultivated land and bare land were mainly found in the soil. For example, in grassland, a large percentage (> 90%) of the total carbon was stored in the soil. This was in accordance with the investigation of Chen et al. [68], where the total carbon stock of the savanna was 204 ± 53 Mg ha^−1^, with 84% below ground and 16% above ground carbon stock. According to Scurlock and Hall [69], soil carbon can store over 75% of the global carbon found in terrestrial ecosystems. Mekuria [66] also found higher carbon stock in soil than other carbon pools for exclosures on communal grazing lands in Ethiopia. However, contrary to the findings of this present study, Girardin et al. [70] and Lü et al. [7] found higher carbon stored in biomass followed by soil and litter in tropical forests.

In the present study, the change in carbon stock caused by change in land cover type was assessed using the area of each land cover type and their corresponding carbon stock values. The study revealed an increase in carbon stock between 1985 and 2000 and a decrease between 2000 and 2016. The change in forest management approach and strategies contributed to the changes of the carbon stock overtime. In 1991, there was a change in natural resource management approach from state forest management to participatory forest management that included intensive soil and water conservation, exclosure establishment and community participation which gave the forest a recovery time for which some improvements in carbon stock have been observed between 1985 and 2000 [33]. Forest expansion and growth increase carbon stock. This was confirmed by a study of Fang et al. [71] who stated carbon storage increased significantly after the late 1970s from 4.38 to 4.75 Pg of carbon by 1998, mainly due to forest expansion and regrowth in China. Silver et al. [72] also indicated that reforestation of abandoned tropical agricultural and pasturelands has the potential to serve as a carbon offset mechanism both above and belowground for at least 40–80 years, and possibly much longer.

However, between 2000 and 2016 a reduction in total carbon stock was recorded due to loss of forest cover caused by encroachment of communities on lands to get wood for fuel, construction materials, more arable land and animal feed. Forestland is a collection of native tree species that has been in existence for quite a long time with many understory vegetation. However, grassland is mainly composed of shrub species with low biomass and total carbon stock as compared to the forest. Consequently, the change from forest to grassland and cultivated land significantly affects total carbon stocks. Our study illustrated that total carbon stock was affected by the land cover change in Wujig Mahgo Waren forest.

In agreement with the present study, previous studies have shown that land cover change is a key factor in carbon stock changes. For example, Shrestha et al. [73] observed a net gain in carbon stock in the larger parts of the mountain watershed in Nepal from 1976 to 1989, while a net loss was recorded in the period between 1989 and 2003. Kashaigili and Majaliwa [16] also realized a reduction in carbon stock from the year 1980 to 2010 in two forests of Tanzania due to forest cover change. Similarly, Gond et al. [74] also reported a 30% loss in carbon stock from 1984 to 2012 in wood-fuel supply basin of Kinshasa. Furthermore, Gaston et al. [44] showcased a loss in above ground carbon stock by 6.6 Pg due to forest degradation in tropical Africa between 1980 and 1990. In the same period [44], recorded 30 Tg loss of above ground carbon due to deforestation and degradation in Ethiopia. A study by Zhang et al. [75] in China showed that carbon stock reduced by 60 Tg between 1995 and 2010 due to land cover change.

### Linkage between soil organic carbon stock and above ground vegetation properties

Various studies have shown that vegetation variability determines topsoil carbon variability in the Savanna and woodland ecosystems [76–78]. In the present study, soil organic carbon and above ground vegetation properties had a positive link, showing that vegetation parameters do appear to be predictors of soil organic carbon stock. Moreover, above ground vegetation parameters such as tree density, DBH and height explained 59% of the variance in soil organic carbon. In a similar study by Li et al. [79] above ground vegetation parameters such as tree height, above ground biomass and tree density elucidated 80% of the variance in soil organic carbon in cold-temperate mountainous forests of Japan. Dar and Sundarapandian [80] also indicated that above ground vegetation properties are common predictors to estimate soil organic carbon stock in complex mountainous forests across different spatial scales. Furthermore, Woollen et al. [81] found the strongest correlation between soil carbon and large tree above ground carbon stocks with 24% of soil carbon variability explained by above ground carbon stock. A study by Kurgat et al. [82] showed that vegetation cover explained 89% of the variability in soil organic carbon in the rangelands of northern Kenya. Similarly, a study by Liu et al. [83] in the Qinghai–Tibetan Plateau China showed a significant correlation between above ground biomass and soil organic carbon. Contrary to this present study, Zhang et al. [84] found that plant biomass, woody plant density and tree height did not emerge as significant predictor variables for soil organic carbon in the subalpine coniferous forest in Southwest China. Mathew et al. [85] also found a poor correlation between soil organic carbon stock and above ground carbon in Mount Kilimanjaro, Tanzania. The inconsistency between these studies shows that environmental factors affecting the distributions of vegetation and soil carbon stocks are site-specific.

Findings from this study show that vegetation parameters can be valuable when predicting soil organic carbon stock in the dry Afromontane forests. This is vital for estimating soil carbon stock, particularly in inaccessible landscapes, as above ground vegetation properties are moderately simple to assess and can be quickly surveyed through remote sensing methods.

## Conclusions

The present study discussed the variation in carbon stock when forest cover changes. There was high variability in total carbon stocks among land cover types with high carbon stocks observed in dense forest and low carbon stocks in cultivated land and bare land. Open forest and grassland sites showed intermediate carbon stock values. However, soil organic carbon did not show significant differences among land cover types. Significantly highest carbon stock was observed in soil carbon pools as compared to the carbon in biomass and litter carbon pools in all land use and land cover types. Land cover change has an impact on carbon stock, with carbon stock slightly increasing between 1985 and 2000, and decreasing from 2000 to 2016. Furthermore, there was a significant correlation between aboveground vegetation properties and soil organic carbon. The aboveground vegetation properties could be useful in the estimation of the soil organic carbon stock in the dry Afromontane forests. Our study indicates that, dry Afromontane forests have the potential to store large amounts of carbon in its biomass and soil. Therefore, management opportunities for increasing biomass can be beneficial for climate mitigation. Furthermore, in this study we tried to analyze the effect of land cover change on carbon stock, however further studies should be conducted on the effect of other biophysical factors on carbon stock.

## Additional file

**Additional file 1.** **Sheet 1.** Aboveground vegetation properties and soil organic carbon data. **Sheet 2.** Total carbon data for all land cover types. **Sheet 3.** Vegetation parameters data for individual species.

## References

[1] Agrawal A, Cashore B, Hardin R, Shepherd G, Benson C, Miller D (2013). Economic contributions of forests.

[2] Chao S (2012). Forest peoples: numbers across the world.

[3] Kumar R, Nandy S, Agarwal R, Kushwaha SPS (2014). Forest cover dynamics analysis and prediction modeling using logistic regression model. Ecol Indic.

[4] Costanza R, d’Arge R, De Groot R, Faber S, Grasso M, Hannon B (1997). The value of the world’s ecosystem services and natural capital. Nature.

[5] Herzog H, Golomb D (2004). Carbon capture and storage from fossil fuel use. Encycl Energy.

[6] FAO (2015). Global forest resources assessment 2015: How are the world’s forests changing?.

[7] Lü X-T, Yin J-X, Jepsen MR, Tang J-W (2010). Ecosystem carbon storage and partitioning in a tropical seasonal forest in Southwestern China. For Ecol Manag.

[8] Thurner M, Beer C, Santoro M, Carvalhais N, Wutzler T, Schepaschenko D (2014). Carbon stock and density of northern boreal and temperate forests. Glob Ecol Biogeogr.

[9] Watson RT, Noble IR, Bolin B, Ravindranath N, Verardo DJ, Dokken DJ (2000). Land use, land-use change and forestry. A special report of the intergovernmental panel on climate change (IPCC).

[10] Gómez DR, Watterson JD, Americano BB, Ha C, Marland G, Matsika E (2006). IPCC guidelines for national greenhouse gas inventories.

[11] Le Quere C, Raupach MR, Canadell JG, Marland G (2009). Trends in the sources and sinks of carbon dioxide. Nat Geosci.

[12] van der Werf GR, Morton DC, DeFries RS, Olivier JGJ, Kasibhatla PS, Jackson RB (2009). CO_2_ emissions from forest loss. Nat Geosci.

[13] Andriamananjara A, Hewson J, Razakamanarivo H, Andrisoa RH, Ranaivoson N, Ramboatiana N (2016). Land cover impacts on aboveground and soil carbon stocks in Malagasy rainforest. Agric Ecosyst Environ.

[14] Mekuria W, Veldkamp E, Haile M, editors. Carbon stock changes with relation to land use conversion in the lowlands of Tigray, Ethiopia. In: Proceedings of Conference on International Research on Food Security, Natural Resource Management and Rural Development; Proceedings of the 36th Meeting of the Italian Society of Agronomy. 2009.

[15] Cui G, Chen Y, Cao Y (2015). Temporal-spatial pattern of carbon stocks in forest ecosystems in Shaanxi, Northwest China. PLoS ONE.

[16] Kashaigili JJ, Majaliwa AM (2010). Integrated assessment of land use and cover changes in the Malagarasi river catchment in Tanzania. Phys Chem Earth.

[17] Sandker M, Nyame SK, Förster J, Collier N, Shepherd G, Yeboah D (2010). REDD payments as incentive for reducing forest loss. Conserv Lett.

[18] MEA (2005). Ecosystems and human well-being: biodiversity Synthesis.

[19] Chidumayo E, Marunda C. Dry forests and woodlands in sub-Saharan Africa: context and challenges. In: Chidumayo EN, Gumbo DJ, editors. The dry forests and woodlands of Africa: managing for products and services. 2010. p. 1–10.

[20] Day M, Gumbo D, Moombe KB, Wijaya A, Sunderland T (2014). Zambia Country Profile: monitoring, reporting and verification for REDD+.

[21] SÁNchez-Azofeifa GA, Kalacska M, Quesada M, Calvo-Alvarado JC, Nassar JM, RodrÍGuez JP (2005). Need for integrated research for a sustainable future in tropical dry forests. Conserv Biol..

[22] Bullock SH (1995). Seasonally dry tropical forests.

[23] Murphy PG, Lugo AE (1986). Ecology of tropical dry forest. Annu Rev Ecol Syst.

[24] Teketay D (1996). Seed ecology and regeneration in dry Afromontane forests of Ethiopia.

[25] GIZ (2015). Land use land cover mapping of PFM project areas and adjacent SLMP watersheds in three regions of Ethiopia.

[26] Wubet T, Kottke I, Teketay D, Oberwinkler F (2003). Mycorrhizal status of indigenous trees in dry Afromontane forests of Ethiopia. For Ecol Manag.

[27] Tesema AB, Ann B, Bo T. Useful trees and shrubs for Ethiopia: identification, propagation and management for agricultural and pastoral communities. 1993.

[28] Asfaw A, Lemenih M, Kassa H, Ewnetu Z (2013). Importance, determinants and gender dimensions of forest income in eastern highlands of Ethiopia: the case of communities around Jelo Afromontane forest. For Policy Econ.

[29] Solomon N, Birhane E, Tadesse T, Treydte AC, Meles K (2017). Carbon stocks and sequestration potential of dry forests under community management in Tigray, Ethiopia. Ecol Process.

[30] Price M, Gratzer G, Alemayehu Duguma L, Kohler T, Maselli D (2011). Mountain forests in a changing world: realizing values, addressing challenges.

[31] Tesfaye G, Teketay D, Fetene M, Beck E (2010). Regeneration of seven indigenous tree species in a dry Afromontane forest, southern Ethiopia. Flora.

[32] Amanuel Z, Girmay G, Atkilt G (2015). Characterisation of agricultural soils in Cascape intervention woredas in southern Tigray, Ethiopia.

[33] Solomon N, Hishe H, Annang T, Pabi O, Asante I, Birhane E (2018). Forest cover change, key drivers and community perception in Wujig Mahgo Waren forest of northern Ethiopia. Land.

[34] Singh J, Dhillon SS (2004). Agricultural geography.

[35] Deng X, Huang J, Rozelle S, Uchida E (2006). Cultivated land conversion and potential agricultural productivity in China. Land Use Policy.

[36] Pearson T, Walker S, Brown S (2005). Sourcebook for land use, land-use change and forestry projects.

[37] Chave J, Réjou-Méchain M, Búrquez A, Chidumayo E, Colgan MS, Delitti WBC (2014). Improved allometric models to estimate the aboveground biomass of tropical trees. Glob Change Biol.

[38] Cairns AM, Brown S, Helmer HE, Baumgardner AG (1977). Root biomass allocation in the world’s upland forests. Oecologia.

[39] Ubuy MH, Gebrehiwot K, Raj AJ (2014). Biomass estimation of exclosure in the Debrekidan watershed, Tigray region, northern Ethiopia. Int J Agric For.

[40] Cleemput S, Muys B, Kleinn C, Janssens MJ. Biomass estimation techniques for enclosures in a semi-arid area: a case study in Northern Ethiopia. University of Göttingen, Germany [www document]. 2004. http://www.tropentag.de/2004/abstracts/full/3.pdf. Accessed 19 Dec 2017.

[41] WBISPP (2000). Manual for woody biomass inventory.

[42] Walkley A, Black IA (1934). An examination of the degtjareff method for determining soil organic matter, and a proposed modification of the chromic acid titration method. Soil Sci.

[43] Gibbs HK, Brown S, Niles JO, Foley JA (2007). Monitoring and estimating tropical forest carbon stocks: making REDD a reality. Environ Res Lett.

[44] Gaston G, Brown S, Lorenzini M, Singh KD (1998). State and change in carbon pools in the forests of tropical Africa. Glob Change Biol.

[45] Kashaigili J, Mdemu M, Nduganda A, Mbilinyi B (2013). Integrated assessment of forest cover change and above-ground carbon stock in Pugu and Kazimzumbwi forest reserves, Tanzania. Adv Remote Sens.

[46] Du H, Zhou G, Fan W, Ge H, Xu X, Shi Y (2010). Spatial heterogeneity and carbon contribution of aboveground biomass of moso bamboo by using geostatistical theory. Plant Ecol.

[47] Rajput BS, Bhardwaj DR, Pala NA (2017). Factors influencing biomass and carbon storage potential of different land use systems along an elevational gradient in temperate northwestern Himalaya. Agrofor Syst.

[48] Mekuria W, Yami M (2013). Changes in woody species composition following establishing exclosures on grazing lands in the lowlands of northern Ethiopia. Afr J Environ Sci Technol.

[49] Tiessen H, Feller C, Sampaio EVSB, Garin P (1998). Carbon sequestration and turnover in semiarid Savannas and dry forest. Clim Change.

[50] Cavanaugh KC, Gosnell JS, Davis SL, Ahumada J, Boundja P, Clark DB (2014). Carbon storage in tropical forests correlates with taxonomic diversity and functional dominance on a global scale. Glob Ecol Biogeogr.

[51] Feyissa A, Soromessa T, Argaw M (2013). Forest carbon stocks and variations along altitudinal gradients in Egdu forest: implications of managing forests for climate change mitigation. Sci Technol Arts Res J.

[52] Watson C, Mourato S, Milner-Gulland EJ (2013). Uncertain emission reductions from forest conservation: REDD in the Bale Mountains, Ethiopia. Ecol Soc.

[53] Chinasho A, Soromessa T, Bayable E (2015). Carbon stock in woody plants of Humbo forest and its variation along altitudinal gradients: the case of Humbo district, Wolaita zone, southern Ethiopia. Int J Environ Prot Policy.

[54] Descheemaeker K, Muys B, Nyssen J, Poesen J, Raes D, Haile M (2006). Litter production and organic matter accumulation in exclosures of the Tigray highlands, Ethiopia. For Ecol Manag.

[55] Ordóñez JAB, de Jong BHJ, García-Oliva F, Aviña FL, Pérez JV, Guerrero G (2008). Carbon content in vegetation, litter, and soil under 10 different land-use and land-cover classes in the central highlands of Michoacan, Mexico. For Ecol Manag.

[56] Aman H. Estimation of carbon stock in dry evergreen montane forest and its role in climate change mitigation: the case of Bale mountains national park, Ethiopia, Addis Ababa University. 2015.

[57] Tesfaye M, Bravo F, Ruiz-Peinado R, Pando V, Bravo-Oviedo A (2016). Impact of changes in land use, species and elevation on soil organic carbon and total nitrogen in Ethiopian Central Highlands. Geoderma.

[58] Haghdoost N, Akbarinia M, Hosseini SM (2013). Land-use change and carbon stocks: a case study, Noor County, Iran. J For Res.

[59] Guo LB, Gifford RM (2002). Soil carbon stocks and land use change: a meta analysis. Glob Change Biol.

[60] Dalal RC, Chan KY (2001). Soil organic matter in rainfed cropping systems of the Australian cereal belt. Soil Res.

[61] Lemenih M, Itanna F (2004). Soil carbon stocks and turnovers in various vegetation types and arable lands along an elevation gradient in southern Ethiopia. Geoderma.

[62] Brown S, Lugo AE (1982). The storage and production of organic matter in tropical forests and their role in the global carbon cycle. Biotropica.

[63] Post W, Emanuel W, Zinke P, Stangenberger A (1982). Soil carbon pools and world life zones. Nature.

[64] Girmay G, Singh BR (2012). Changes in soil organic carbon stocks and soil quality: land-use system effects in northern Ethiopia. Acta Agric Scand Sect B Soil Plant Sci.

[65] Girmay G, Singh BR, Mitiku H, Borresen T, Lal R (2008). Carbon stocks in Ethiopian soils in relation to land use and soil management. Land Degrad Dev.

[66] Mekuria W (2013). Changes in regulating ecosystem services following establishing exclosures on communal grazing lands in Ethiopia: a synthesis. J Ecosyst.

[67] Simegn TY, Soromessa T, Bayable E (2014). Forest carbon stocks in lowland area of Simien Mountains National Park: implication for climate change mitigation. Sci Technol Arts Res J.

[68] Chen X, Hutley LB, Eamus D (2003). Carbon balance of a tropical savanna of northern Australia. Oecologia.

[69] Scurlock JMO, Hall DO (1998). The global carbon sink: a grassland perspective. Glob Change Biol.

[70] Girardin CAJ, Malhi Y, AragÃO LEOC, Mamani M, Huaraca Huasco W, Durand L (2010). Net primary productivity allocation and cycling of carbon along a tropical forest elevational transect in the Peruvian Andes. Glob Change Biol.

[71] Fang J, Chen A, Peng C, Zhao S, Ci L (2001). Changes in forest biomass carbon storage in China between 1949 and 1998. Science.

[72] Silver WL, Ostertag R, Lugo AE (2000). The potential for carbon sequestration through reforestation of abandoned tropical agricultural and pasture lands. Restor Ecol.

[73] Shrestha BM, Dick ØB, Singh B (2010). Effects of land-use change on carbon dynamics assessed by multi-temporal satellite imagery in a mountain watershed of Nepal. Acta Agric Scand Sect B Soil Plant Sci.

[74] Gond V, Dubiez E, Boulogne M, Gigaud M, Peroches A, Pennec A (2016). Forest cover and carbon stock change dynamics in the Democratic Republic of Congo: case of the wood-fuel supply basin of Kinshasa. Bois et Forêts des Tropiques.

[75] Zhang M, Huang X, Chuai X, Yang H, Lai L, Tan J (2015). Impact of land use type conversion on carbon storage in terrestrial ecosystems of China: a spatial-temporal perspective. Sci Rep.

[76] Bird MI, Veenendaal EM, Moyo C, Lloyd J, Frost P (2000). Effect of fire and soil texture on soil carbon in a sub-humid savanna (Matopos, Zimbabwe). Geoderma.

[77] Rossi J, Govaerts A, De Vos B, Verbist B, Vervoort A, Poesen J (2009). Spatial structures of soil organic carbon in tropical forests—a case study of southeastern Tanzania. Catena.

[78] Wang L, Okin GS, Caylor KK, Macko SA (2009). Spatial heterogeneity and sources of soil carbon in southern African savannas. Geoderma.

[79] Li P, Wang Q, Endo T, Zhao X, Kakubari Y (2010). Soil organic carbon stock is closely related to aboveground vegetation properties in cold-temperate mountainous forests. Geoderma.

[80] Dar J, Sundarapandian S (2013). Soil organic carbon stock assessment in two temperate forest types of western Himalaya of Jammu and Kashmir, India. For Res.

[81] Woollen E, Ryan CM, Williams M (2012). Carbon stocks in an African woodland landscape: spatial distributions and scales of variation. Ecosystems.

[82] Kurgat BK, Golicha D, Giese M, Kuria SG, Asch F. Relationship between vegetation cover types and soil organic carbon in the rangelands of Northern Kenya. Livestock Res Rural Dev. 2014;26. http://www.lrrd.org/lrrd26/9/kurg26162.html. Retrieved 21 Oct 2017.

[83] Liu W, Chen S, Qin X, Baumann F, Scholten T, Zhou Z (2012). Storage, patterns, and control of soil organic carbon and nitrogen in the northeastern margin of the Qinghai–Tibetan Plateau. Environ Res Lett.

[84] Zhang Y, Duan B, Xian J, Korpelainen H, Li C (2011). Links between plant diversity, carbon stocks and environmental factors along a successional gradient in a subalpine coniferous forest in Southwest China. For Ecol Manag.

[85] Mathew MM, Majule AE, Sinclair F, Marchant R (2016). Relationships between on-farm tree stocks and soil organic carbon along an altitudinal gradient, Mount Kilimanjaro, Tanzania. For Trees Livelihoods.

